# Time-ordered dysregulated ceRNA networks reveal disease progression and diagnostic biomarkers in ischemic and dilated cardiomyopathy

**DOI:** 10.1038/s41420-021-00687-7

**Published:** 2021-10-16

**Authors:** Ziyi Bai, Haoran Sun, Xiuhong Li, Jie Wu, Hao Yuan, Guangde Zhang, Haixiu Yang, Hongbo Shi

**Affiliations:** 1 College of Bioinformatics Science and Technology, Harbin Medical University, Harbin, China; 2 Laboratory of Medical Genetics, Harbin Medical University, Harbin, China; 3 Department of Cardiology, The Fourth Affiliated Hospital of Harbin Medical University, Harbin, China

**Keywords:** Gene expression, Gene expression profiling

## Abstract

Ischemic cardiomyopathy (ICM) and dilated cardiomyopathy (DCM) are the two main causes of heart failure (HF). Despite similar clinical characteristics and common “HF pathways”, ICM and DCM are expected to have different personalized treatment strategies. The underlying mechanisms of ICM and DCM have yet to be fully elucidated. The present study developed a novel computational method for identifying dysregulated long noncoding RNA (lncRNA)–microRNA (miRNA)–mRNA competing endogenous RNA (ceRNA) triplets. Time-ordered dysregulated ceRNA networks were subsequently constructed to reveal the possible disease progression of ICM and DCM based on the method. Biological functional analysis indicated that ICM and DCM had similar features during myocardial remodeling, whereas their characteristics differed during progression. Specifically, disturbance of myocardial energy metabolism may be the main characteristic during DCM progression, whereas early inflammation and response to oxygen are the characteristics that may be specific to ICM. In addition, several panels of diagnostic biomarkers for differentiating non-heart failure (NF) and ICM (NF-ICM), NF-DCM, and ICM-DCM were identified. Our study reveals biological differences during ICM and DCM progression and provides potential diagnostic biomarkers for ICM and DCM.

## Introduction

Ischemic cardiomyopathy (ICM) and dilated cardiomyopathy (DCM) are the two most commonly occurring etiologies for heart failure (HF). Despite similar clinical characteristics, ICM and DCM respond differently to the same medication and ICM has a worse prognosis [1]. Nowadays, the understanding of ICM and DCM remains incomplete. Moreover, due to strong similarities between the two diseases, the clinical differentiation between ICM and DCM currently depends mainly on the results of coronary angiography, a procedure that is both invasive and expensive [2].

Currently, great effort is being made to investigate common HF pathways and disease-specific characteristics in ICM and DCM [3–11]. At the transcription level, for one thing, microarray gene expression profiles of myocardial samples and peripheral blood mononuclear cells have been confirmed to be able to distinguish ICM from DCM [3, 5, 6]. A study reported that the significantly differentially expressed (SDE) genes shared in common between ICM and DCM were mainly involved in cell proliferation and signal transduction, whereas the uniquely expressed genes of ICM usually had catalytic activity, and those of DCM were frequently involved in metabolism [4]. For another, sequencing-based data have been applied to analyze the expression levels of myocardial mRNAs, microRNAs (miRNAs), and long noncoding RNAs (lncRNAs) in failing human hearts, and research revealed that the expression profiles of lncRNAs had a higher classification capability for failing hearts of different pathologies compared with mRNAs and miRNAs [7]. At the proteome and metabolome levels, gender-specific pathways in ICM and DCM have been unveiled in myocardial samples [10]. In the same period, commonalities of HF pathways and disease-specific metabolic features were founded through the analysis of plasma samples, and metabolite biomarkers for differentiating patients with ICM and DCM were identified [11]. However, the underlying biological mechanisms of ICM and DCM contributing to HF have yet to be fully elucidated, and the above-mentioned studies were all performed at the single molecular level, ignoring the interactions among molecules.

Both theoretical and experimental studies have demonstrated that different types of RNA transcripts contain numerous miRNA binding sites, and they can communicate with and regulate each other through competing for shared miRNAs, thus acting as competing endogenous RNAs (ceRNAs) [12, 13]. LncRNAs could compete with miRNA target mRNAs for miRNAs, and thus realizing mutual regulation [13]. This type of ceRNA has been reported in cardiovascular diseases. For example, lncRNA NONMMU022555 contributes to cardiac fibrosis via acting as a ceRNA of let-7d, as determined through a combination of in vitro and in vivo studies on myocardial infarction hearts of mice [14]. LncRNA CYTOR might function as a ceRNA of miR-155 to counteract miR-155-mediated repression of IKBKE, and played a protective role in mice cardiac hypertrophy [15]. However, the potential crosstalk of ceRNAs in ICM and DCM has yet to be systematically investigated.

**Fig. 1:**
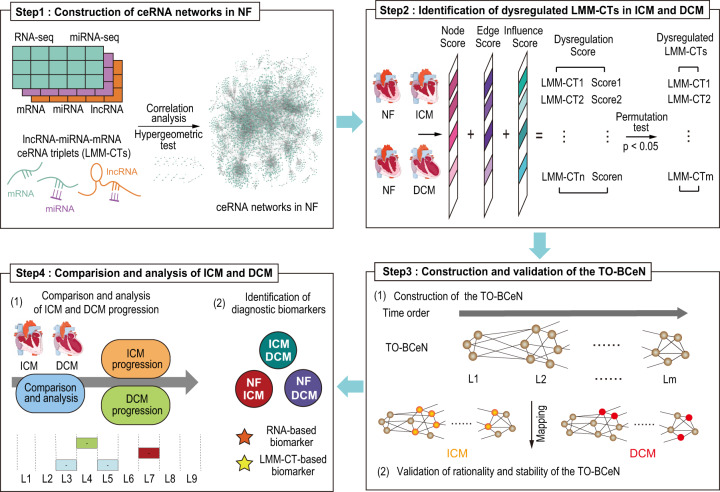
Workflow of the present study. Step 1: CeRNA networks in NF were constructed using sample-matched RNA-seq and miRNA-seq data and experimentally supported interaction information. Step 2: Dysregulated LMM-CTs in ICM and DCM were identified applying our method, which integrated the dysregulation extent of gene expression, gene interactions, and the influence of the dysregulated LMM-CTs. Step 3: TO-BCeN in NF was constructed based on the breadth-first algorithm. After mapping dysregulated LMM-CTs of ICM and DCM to the TO-BCeN, its rationality and stability were verified. Step 4: The disease progressions of ICM and DCM were compared based on the TO-BCeN, and diagnostic biomarkers for ICM and DCM were identified.

In the present study, dysregulated lncRNA–miRNA–mRNA ceRNA triplets (LMM-CTs) in ICM and DCM were systematically identified, and time-ordered dysregulated ceRNA networks for the two diseases were constructed. The workflow is shown in Fig. 1. Characteristics of the disease progressions between ICM and DCM were also compared, and several panels of diagnostic biomarkers associated with ICM and DCM were identified.

## Results

### Dysregulated LMM-CTs in ICM and DCM

Using sample-matched RNA-seq and miRNA-seq data, the method developed in this study was applied to identify dysregulated LMM-CTs in ICM and DCM. As a result, 1271 dysregulated LMM-CTs including 97 lncRNAs, 85 miRNAs, and 675 mRNAs in ICM were obtained, and 1298 dysregulated LMM-CTs consisting of 107 lncRNAs, 96 miRNAs, and 727 mRNAs in DCM were identified (Supplementary Table S1).

Subsequently, the proportions of SDE genes and the known disease-associated genes (DisGenes) in dysregulated LMM-CTs were respectively investigated. SDE genes were selected using DESeq2 with *p* < 0.01 and |log_2_ fold change (FC) | > 1.2. The results showed that the proportions of SDE genes and DisGenes in dysregulated LMM-CTs were both significantly higher than that of NF LMM-CTs (hypergeometric test, SDE genes: *p* = 5.71 × 10^−3^ for ICM, and *p* = 8.30 × 10^−3^ for DCM; and DisGene: *p* = 1.57 × 10^−2^ for ICM, and *p* = 5.02 × 10^−3^ for DCM).

### Time-ordered background ceRNA networks (TO-BCeN) in NF

To construct TO-BCeN in NF, hsa-miR-3615 and hsa-miR-580-3p were chosen as the initial nodes because these two miRNAs were both in the top 10% lowest dysregulated score in ICM and DCM (for details, see Materials and methods). The TO-BCeN was then obtained using the breadth-first search (BFS) algorithm, and single gene-based networks were then converted into LMM-CT-based networks.

**Fig. 2:**
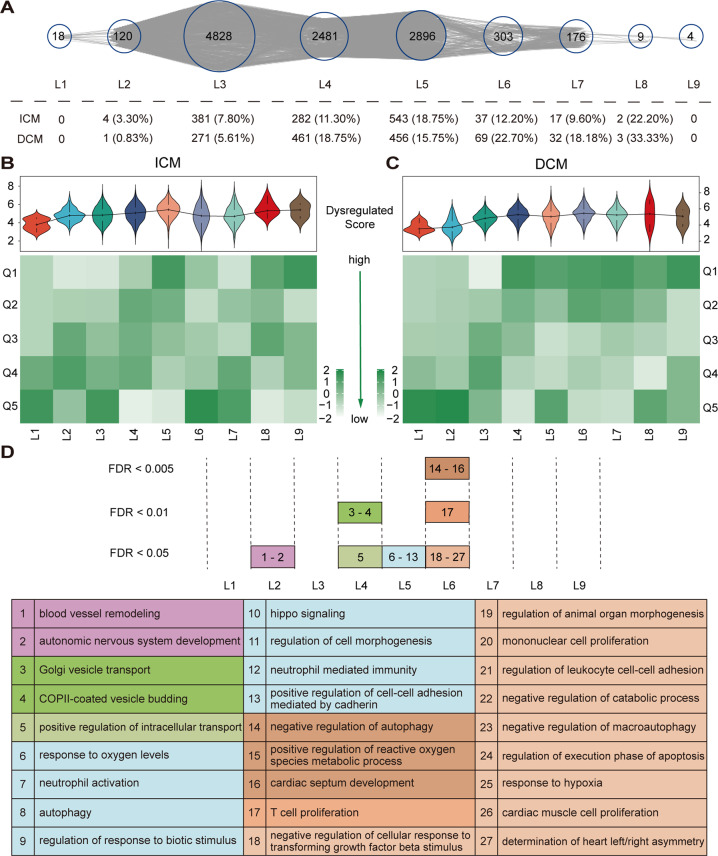
The constructed TO-BCeN and validation of its rationality. **A** Global landscape of the TO-BCeN and summary of dysregulated LMM-CTs for ICM and DCM in the TO-BCeN. L1–L9 represents time-ordered levels for the TO-BCeN. The numbers presented in circles indicate the number of LMM-CTs at a particular level. The numbers inside and outside of the parentheses denote the number and proportion of dysregulated LMM-CTs at the corresponding level, respectively. **B**, **C** The violin diagrams and heatmaps of the dysregulated score distribution of LMM-CTs among nine time-ordered levels for ICM and DCM, respectively. For each level in the heatmap, dysregulated scores were divided equally into five parts, and the number of LMM-CTs in each part was computed. The color of the square denotes the corresponding standard score. **D** Significantly enriched biological functions for mRNAs in ICM dysregulated LMM-CTs at each time-ordered level are shown. The numbers in the plot (Top) corresponded to the index number of significantly enriched functions listed in the table (Bottom). The pink, green, blue, and orange colors represent the stages of vascular dysfunction, signal transport, responses after myocardial ischemia and myocardial remodeling, respectively.

The TO-BCeN consisted of 9 time-ordered levels (denoted L1–L9). As shown in Fig. 2A and Supplementary Table S2, the dysregulated LMM-CTs of ICM and DCM were then respectively mapped to the TO-BCeN. The results revealed that the majority of the dysregulated LMM-CTs were distributed in L3–L7 levels.

### Validation of rationality and stability of the TO-BCeN

When constructing the TO-BCeN, it was assumed that its initial nodes should be relatively stable, which would otherwise cause the instability of the entire TO-BCeN. Therefore, the distribution of dysregulated scores of LMM-CTs among nine time-ordered levels for ICM and DCM were respectively examined (Supplementary Table S2). As shown in Fig. 2B, C, as the time-ordered level increased, the dysregulated scores of LMM-CTs for DCM gradually increased, a finding that was consistent with our hypothesis. ICM presented the same phenomenon, with the exception of L6 and L7 levels.

ICM has a clear causal precipitant (myocardial ischemia). Taking ICM for example, the rationality of the TO-BCeN was validated from the perspective of its biological functions. For ICM-dysregulated LMM-CTs at each time-ordered level, based upon the mRNAs at those levels, significantly enriched Gene Ontology biological process (GO BP) terms were acquired with false discovery rate < 0.05 using the R package “clusterProfiler” (Supplementary Table S3).

To clearly illustrate our results, significantly enriched GO BP terms typically associated with ICM are partially shown in Fig. 2D. At L2 time-ordered level, functions associated with vascular dysfunction were significantly enriched, including “blood vessel remodeling” and “autonomic nervous system development”. Vascular dysfunction was able to promote plaque development, leading to coronary stenosis and the formation of coronary artery disease [16]. By contrast, functional categories associated with signal transport were overrepresented at L4. At the L5 level, functions associated with responses after myocardial ischemia were found to be significantly enriched, including the “response to oxygen levels”, “neutrophil activation”, “regulation of response to biotic stimulus”, and “autophagy”. Inflammation and autophagy are known to play crucial roles in myocardial ischemia [17], and neutrophils participate in a series of pathophysiological processes following ischemia [18]. Compared with L2–L5, mRNAs at L6 mainly functioned in processes relevant to myocardial remodeling. The overrepresented functional categories included “autophagy”, “regulation of transforming growth factor-β (TGF-β)”, “mononuclear cell and cardiac muscle cell proliferation”, “regulation of leukocytes”, and “macroautophagy and lymphocyte differentiation”. The myocardial remodeling process usually includes the removal of dead cardiomyocytes and the activation of cardiac fibroblasts into myofibroblasts, which trigger inflammatory and immune reactions [19]. Taken together, the results indicated that the TO-BCeN we constructed offered a reasonably good reflection of the state of ICM progression.

In this study, hsa-miR-3615 and hsa-miR-580-3p were chosen as the initial nodes to construct the TO-BCeN. To test the stability of the TO-BCeN, two other genes at L1 level of the original TO-BCeN were randomly selected as initial nodes, and the differences in each level for the new TO-BCeN were calculated compared with the original one. This procedure was repeated 10 times, and the results are shown in Table 1. It was found that both the means and standard deviations (SDs) of the overall level changes for new TO-BCeNs were very small. It was also noted that the majority of the changes manifested at the previous and the following time-ordered levels, which may be due to the fact that the constructed TO-BCeN was not single node-based, but was an LMM-CTs-based network. These results demonstrated that the original TO-BCeN was stable.

**Table 1:** Summary of level order changes with different initial nodes.

Initial node	No. of not changed	No. of cross 1 level	No. of cross 2 levels	No. of cross multiple levels	Mean of level change	SD of level change
ADARB1,MIRLET7BHG	2311	4344/−4180	0	0	0.015	0.886
HMGCS1,RPL15	1065	8135/−1635	0	0	0.599	0.736
HMGCS1,MIRLET7BHG	970	8419/−1310	0/−66	0/−65	0.622	0.779
HMGCS1,THUMPD3-AS1	918	8699/−1218	0	0	0.69	0.662
GIGYF1,THUMPD3-AS1	2287	4817/−3668	0/−41	0/−22	0.092	0.899
HMGCS1,LUC7L3	1065	8135/−1635	0	0	0.6	0.736
MAT2B,MIRLET7BHG	2843	1803/−6189	0	0	−0.405	0.757
MAT2B,THUMPD3-AS1	2004	2587/−6176	0/−46	0/−22	−0.346	0.852
MIRLET7BHG,ZBTB39	2853	4334/−3648	0	0	0.063	0.856
MIRLET7BHG,THUMPD3-AS1	2786	3619/−4430	0	0	−0.074	0.858

Note: For a node, the positive and negative number denotes its level changes to the previous or the next level, respectively, in the newly generated TO-BCeN contra the original one. ‘Zero’ denotes that there was no change between the two TO-BCeNs. If a node belonged to level 3 in the original TO-BCeN and is classified into level 2 in the new TO-BCeN, then the change number is considered as −1. Multiple levels indicate that the level change spans more than two levels. *No.* the abbreviation of number, *SD* standard deviation.

### Comparison and analysis of ICM and DCM progression

**Fig. 3:**
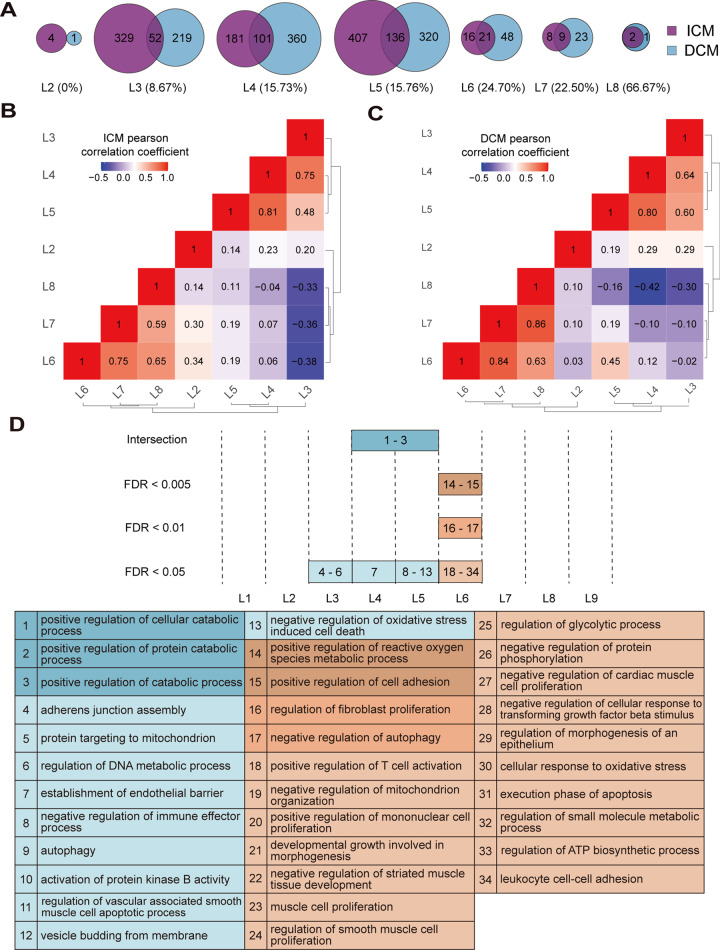
Comparison and analysis of ICM and DCM progression. **A** The number and the proportion of common dysregulated LMM-CTs at each time ordered level for ICM and DCM. The number in parentheses denotes the proportion of common dysregulated LMM-CTs at that level. **B**, **C** The hierarchical clustering of the PCC among different time-ordered levels for ICM and DCM, respectively. **D** The overrepresented functions using mRNAs in DCM dysregulated LMM-CTs at each time-ordered level. The numbers in the plot (Top) correspond to the index number of significantly enriched functions listed in the table (Bottom). The blue and orange colors represent DCM at the progressed and advanced stage, respectively.

The number and the proportion of common dysregulated LMM-CTs at each time-ordered level for ICM and DCM are shown in Fig. 3A. The increases observed in the proportion values suggest that the two diseases might have more similarities in the latter stages. The correlations among different time-ordered levels for ICM and DCM were also examined. For a given level of ICM, the gene set variation analysis (GSVA) was applied to estimate the variation activity of ICM dysregulated LMM-CTs for each sample [20]. Pearson’s correlation coefficient (PCC) was subsequently calculated between any two levels. Unsupervised hierarchical clustering was performed based on the PCC using the R package “pheatmap” according to the Euclidean distance and the complete linkage method. As shown in Fig. 3B, C, both ICM and DCM obtained three clusters, L2, L3–L5, and L6–L8, demonstrating similar disease progress of the two diseases.

It was possible to infer some of the processes for DCM based on the TO-BCeN. Significantly enriched GO BP terms were obtained at each level for DCM (Fig. 3D and Supplementary Table S4). The result revealed that there were no overrepresented biological functions at the L2 level. For the DCM progression stage (corresponding to levels L3–L5), each level was observed to have functions associated with energy metabolism, including processes such as “protein targeting to the mitochondria”, “positive regulation of cellular and protein catabolism”, and the “regulation of lipid catabolism”. This indicated that disturbance of myocardial energy metabolism might be the main characteristic during DCM progression. A previous study demonstrated that disorders of myocardial energy metabolism could lead to impaired myocardial function, thereby causing the initiation of DCM [21]. In addition, the establishment of an endothelial barrier at the L4 level was overrepresented, and functional categories including “immunity”, “autophagy”, “vascular-associated smooth-muscle cell apoptosis”, and “oxidative stress response” were significantly enriched at the L5 level. Similarly to ICM, mRNAs of DCM dysregulated LMM-CTs at L6 level were also significantly enriched in myocardial remodeling-associated functions, including “regulation of fibroblast proliferation”, “cardiac muscle cell proliferation”, “lymphocyte activation”, “autophagy”, “leukocyte cell–cell adhesion”, and “regulation of the TGF-β signaling pathway”.

Simultaneously, differences between ICM and DCM were identified. Certain functional categories associated with early inflammation of myocardial ischemia were overrepresented at ICM L5 level, including “neutrophil activation” and “neutrophil-mediated immunity”. In addition, the “response to oxygen” was overrepresented at ICM L5 level. But functions associated with inflammation and oxygen were not detected at DCM L5 level. At present, no evidence has been gathered to support that myocardium in non-ischemic HF experiences oxygen limitation [22].

### Potential ICM and DCM diagnostic biomarkers

Based on the identified dysregulated LMM-CTs in ICM and DCM, two types of diagnostic biomarkers were identified. One was for distinguishing ICM/DCM patients from the NF controls (denoted as NF-ICM and NF-DCM), and the other was for differentiating ICM from DCM patients (denoted as ICM-DCM).

For the identification of NF-ICM diagnostic biomarkers, 1271 significantly dysregulated ICM LMM-CTs were investigated, including 675 mRNAs, 85 miRNAs, and 97 lncRNAs. By applying our method, optimal NF-ICM biomarkers were then obtained. The results are shown in Table 2; three panels of NF-ICM biomarkers defined by two mRNAs (CCDC113 and ZCCHC3), two miRNAs (hsa-miR-155-5p and hsa-miR-221-3p), and three lncRNAs (KCTD21-AS1, AC010969.2, and AC026356.1) were acquired. Similarly, three panels of NF-DCM biomarkers defined by one mRNA (CHDH), three miRNAs (hsa-miR-222-3p, hsa-miR-301b-3p, and hsa-miR-320b), and three lncRNAs (AC015802.4, HCG11, and LINC01278) were obtained. The accuracies and area under the receiver operating characteristic curve (AUC) values in the training and test set based on leave-one-out cross-validation (LOOCV) are shown in Table 2. This analysis revealed that the identified biomarkers could effectively differentiate patients with ICM/DCM from the controls.

**Table 2:** Classification performance of the identified diagnostic biomarkers associated with ICM and DCM based on LOOCV.

Biomarker	RNA	Combination	Training set (accuracy/AUC)	Test set (accuracy/AUC)
NF-ICM	mRNA	CCDC113 ZCCHC3	0.938/0.984	GSE116250 0.778/0.725 GSE1145 0.738/0.777
	lncRNA	KCTD21-AS1 AC010969.2 AC026356.1	0.875/0.828	GSE116250 0.704/0.610
	miR	hsa-miR-221-3p hsa-miR-155-5p	0.938/0.984	GSE53080 0.952/0.962
NF-DCM	mRNA	CHDH	0.938/1.000	GSE116250 0.843/0.932 GSE1145 0.816/0.721
	lncRNA	LINC01278 HCG11 AC015802.4	0.938/1.000	GSE116250 0.804/0.801
	miR	hsa-miR-222-3p hsa-miR-301b-3p hsa-miR-320b	0.875/0.969	GSE53080 0.897/0.952
ICM-DCM	mRNA	H2AFX FOXK2	0.875/0.844	GSE116250 0.760/0.539 GSE1145 0.466/0.611
	lncRNA	SDCBP2-AS1 LINC01089	0.875/0.875	GSE116250 0.740/0.593
	miR	hsa-miR-545-5p	0.875/0.938	GSE53080 0.618/0.606
	ceRNA	TNRC6A/hsa-miR-3065-3p/OTUD6B-AS1	0.938/0.938	/

The identification of ICM-DCM diagnostic biomarkers underwent the same procedure. As a result, three panels of ICM-DCM biomarkers defined by two mRNAs (H2AFX, FOXK2), one miRNA (hsa-miR-545-5p), and two lncRNAs (LINC01089 and SDCBP2-AS1) were obtained (Table 2). Compared with biomarkers of NF-ICM and NF-DCM, the ICM-DCM biomarkers showed similar classification capabilities in the training set, but a poor performance in the test set.

To further explore the capability of the dysregulated LMM-CTs in distinguishing ICM from DCM samples, a multiple regression model was employed to compute a dysregulated score for each patient for a given dysregulated LMM-CT (for details, see the Materials and methods). In the same way, one LMM-CT including TNRC6A, hsa-miR-3065-3p, and OTUD6B-AS1 was identified, showing both accuracy and an AUC value of 0.938 in the training set using LOOCV.

## Discussion

To investigate the similarities and differences between ICM and DCM based on ceRNA mechanisms, this study proposed a novel computational approach for identifying dysregulated LMM-CTs in ICM and DCM, which integrated the dysregulation extent of gene expression, gene interactions, and the influence of the dysregulated LMM-CTs. The disease progression of both ICM and DCM was subsequently systematically compared and analyzed by constructing time-ordered dysregulated ceRNA networks. Additionally, multiple panels of diagnostic biomarkers associated with ICM and DCM were identified.

Our study has concentrated on the origin and development of the disease. Therefore, ICM and DCM were both compared with NF samples. Time-series gene expression data are capable of providing more valuable information than steady-state, although this type of data for cardiomyopathy was unavailable up to this point. Based on the topological structure of the networks, time-ordered dysregulated ceRNA networks for ICM and DCM were constructed, and the rationality and stability of the networks were verified. This method did not require correction or standardization of expression values among different time points and conditions. Furthermore, the method was able to be combined with time-series sequencing data to reveal even more precise and clear biological processes during disease progression.

Biological functions at each time-ordered level for DCM and ICM were analyzed. Based on the results, shared functions among different levels were not observed. However, certain functions did belong to the same functional category, and so some overlap existed among the time-ordered levels. Simultaneously, it was found that inflammation appeared in the early stage of ICM, whereas DCM did not exhibit this phenomenon. This observation, however, did not mean that the early stages of DCM have nothing to do with inflammation since viral myocarditis could lead to the initiation of DCM. One possible reason may be an absence of inflammation-induced DCM in enrolled patients. Additionally, the NF donor heart samples collected for the present study were taken from individuals who had experienced an acute and ultimately fatal event, and they were therefore different from the normal hearts. Due to the limitations of clinical heart biopsies, transcriptome analysis for the study of HF remains a challenge.

The present study has also presented a multiple regression model for identifying LMM-CT-based biomarkers to distinguish ICM from DCM. However, owing to a lack of sample-matched mRNA, miRNA, and lncRNA expression data of ICM and DCM, the efficiencies of the identified LMM-CT biomarkers in the test set were not clear, and further studies are required. Our study has provided a strategy for identifying multiple types of combinations of molecules as diagnostic biomarkers, which not only considered the expression variation of the molecules but also reflected potential interactions among them. This idea can be applied to other diseases.

In conclusion, we systematically investigated the shared and distinct biological features during ICM and DCM progression by constructing time-ordered dysregulated ceRNA networks and provided a new idea for the identification of multiple types of molecules combined as diagnostic biomarkers. Our results will shed new light on deciphering the underlying pathogenetic mechanisms of ICM and DCM, and provide a basis for developing etiology-specific therapies for HF patients in the future.

## Methods

### Data collection and pre-processing

The sample paired FASTQ format RNA-seq and miRNA-seq data of GSE46224 [7] were downloaded from the ArrayExpress database (https://www.ebi.ac.uk/arrayexpress/), which were derived from left ventricular myocardial tissue. The dataset included eight patients with ICM, eight patients with DCM, and eight NF individuals (Supplementary Table S5). For RNA-seq data, the adapter sequences were removed using Trimmomatic [23] (version 0.36), the RNA annotations were retrieved from GENCODE (release 32), and the sequencing reads were aligned against the human genome (hg38) using STAR [24] (version 2.5.4). Read counts were then obtained using HTSeq-count [25] (version 0.11.2), and transcripts per million (TPM) were used to measure the mRNA and lncRNA expression levels. For miRNA-seq data, the data set was processed using sRNAbench [26]. Reads per million (RPM) were used to measure miRNA expression levels. mRNAs with at least 5 reads, and miRNAs and lncRNAs with at least 1 read, in >50% of the samples were retained for further analysis. SDE genes were obtained using DESeq2 [27].

In addition, FASTQ format RNA-seq data of GSE116250 [9] and miRNA-seq data of GSE53080 [28] were also downloaded from the ArrayExpress database. GSE116250 and GSE53080 contained 13 and 14 ICM samples, 37 and 22 DCM samples, and 14 and 10 NF individuals, respectively. The data were processed using the same pipeline as above, with the exception of the sequencing library protocol of miRNA-seq. The microarray data of GSE1145 were downloaded from the Gene Expression Omnibus database, which included 31 ICM samples, 27 DCM samples, and 11 NF individuals. Probe sets corresponding to multiple gene symbol identifiers were removed, and the expression values of each gene detected by at least two probes were averaged. These three datasets were subsequently used as the test sets (Supplementary Tables S5 and S6).

Experimentally verified miRNA–mRNA interactions were collected from Tarbase [29] (version 8.0), miRTarbase [30] (version 7.0) and miRrecords [31] (version 4). Experimentally supported miRNA–lncRNA regulatory associations were retrieved from the Encyclopedia of RNA Interactomes database (http://starbase.sysu.edu.cn/index.php) and DIANA-LncBase [32] (version 3). An overall total of 758,195 mRNA-miRNA and 114,951 mRNA-lncRNA interactions were acquired.

ICM- and DCM-associated mRNAs were collected from the DisGeNET [33] (version 7.0) database, which combines several currently and widely used gene-disease association databases. ICM- and DCM-associated miRNAs and lncRNAs were retrieved using a comprehensive literature review. Relevant articles were compiled in two ways. One is from HMDD [34] (version 3.2) and LncRNADisease [35] (version 2.0) databases using the search phrases “ischemic cardiomyopathy” and “dilated cardiomyopathy”. And another is from PubMed using the search phrases “ischemic cardiomyopathy AND microRNA”, “dilated cardiomyopathy AND microRNA”, “ischemic cardiomyopathy AND (lncRNA OR long non-coding RNA)”, and “dilated cardiomyopathy AND (lncRNA OR long noncoding RNA)”. Each article was manually searched for miRNAs and lncRNAs that were aberrantly expressed in ICM and DCM. Finally, 103 mRNAs, 9 lncRNAs, 25 miRNAs associated with ICM, and 828 mRNAs, 7 lncRNAs, and 56 miRNAs associated with DCM were obtained.

### Identification of LMM-CTs in NF

In this study, it was assumed that LMM-CTs would exist in NF samples and that their dysfunction would lead to disease initiation and progression. LMM-CTs in NF samples were identified based on ceRNA mechanisms. An LMM-CT was identified if it met all the following criteria: (i) The mRNA and the lncRNA shared a significant number of miRNAs, as determined by a hypergeometric test (*P* < 0.01); and (ii) the PCCs of lncRNA–mRNA, miRNA–mRNA, and miRNA–lncRNA interactions were >0.7, <−0.7, and <−0.7 with a *P*-value < 0.05, respectively. Ultimately, 10,860 LMM-CTs comprising 192 lncRNAs, 155 miRNAs, and 2834 mRNAs in NF samples were obtained. A ceRNA network for the NF samples including these LMM-CTs was thereby constructed.

### Identification of dysregulated LMM-CTs in ICM and DCM

Dysregulated LMM-CTs were identified in ICM and DCM using sample-matched lncRNA, miRNA, and mRNA expression data. A dysregulated LMM-CT was identified by considering the following three factors: (i) The dysregulation extent of gene expression (nodes); (ii) the dysregulation extent of gene interactions (edges); and (iii) the influence of the dysregulation of the LMM-CT on genes that directly interacted with it in the NF ceRNA network.

First, according to the extent of differential expression, the node score of LMM-CT was calculated using Eqs. (1) and (2) [36]

1 $${{DotScore}} = F_Z(z) = P\left\{ {Z \le z} \right\} = 1 - \sqrt {\frac{2}{{\pi \sigma ^2}}} \int _0^\infty e^{ - \left( {x^2/2\sigma ^2 + \lambda z/x} \right)}dx,{{{\mathrm{z}}}} \ge {{{\mathrm{0}}}}$$

2 $$z = \left( { - \log _{10}P} \right) \cdot |\log _2{\rm{FC}}|$$

where FC is the corresponding FC of expression, *F*_*Z*_(z) is the cumulative distribution function (CDF) of *z*-statistics, *λ* = ln10 and *σ*^2^ is the variance of FC, and *P* is the *P*-value reflecting the significance of differential expression calculated by DESeq2.

Secondly, the edge score was computed according to Eqs. (3)–(6)

3 $${{EdgeScore}} = \varphi _{\Pi}\left\{ {Y\cdot [F(r_{\rm{case}}) - F(r_{\rm{control}})]} \right\}$$

4 $$\varphi _{\Pi}(x) = P\left\{ {{\Pi} \le x} \right\} = \frac{1}{{\sigma \sqrt {2\pi } }}\mathop {\int }\nolimits_{ - \infty }^x \exp \left( { - \frac{{(t - u)^2}}{{2\sigma ^2}}} \right)dt, - \infty \,<\, x \,<\, + \infty$$

5 $$F(r) = \frac{1}{2}\ln \left( {\frac{{1 + r}}{{1 - r}}} \right)$$

6 $${{{Y = }}}\left\{ {\begin{array}{*{20}{c}} {{{{\mathrm{1,}}}}} & {r_{\rm{control}} \,<\, 0} \\ {{{{\mathrm{ - 1,}}}}} & {r_{\rm{control}} \,>\, 0} \end{array}} \right.$$

Where $\varphi _{\Pi}\left( x \right)$ is the CDF of the normal distribution. $\mu = {{{Y}}} \cdot ( {\mu _{F(r_{\rm{case}})} - \mu _{F(r_{\rm{control}})}} )$ and $\sigma ^2 = \sigma ^2_{F(r_{\rm{case}})} + \sigma ^2_{F(r_{\rm{control}})}$. *F* is the Fisher transformation function, which makes the data approximately follow the normal distribution [37], and *r*_case_ and *r*_control_ are the PCCs of gene expression in case and control samples, respectively.

Thirdly, the influence score of the dysregulation of an LMM-CT on genes was calculated using Eq. (7)

7 $${{InfluenceScore}} = {{{{IS}}_{{{miR}}|{{lncRNA}}}}} + {{{{IS}}_{{{mRNAlncRNA}}|{{miRNA}}}}} + {{{{IS}}_{{{miR}}|{{mRNA}}}}}$$

where ${{{{IS}}_{{{miR}}|{{lncRNA}}}}}$ denotes the influence score of the lncRNA in the LMM-CT under consideration on miRNAs that directly interact with the lncRNA, and ${{{{IS}}_{{{miR}}|{{lncRNA}}}}}$ is defined as 1 − (*P*-value), where the *P*-value is computed using Fisher’s exact test, which reflects the significance level of the miRNAs directly interacting with the lncRNAs that are enriched in SDE miRNAs (*P* < 0.01 and |log2FC | >1.2). Similarly, *IS*_*mRNAlncRNA|miRNA*_ and *IS*_*miR|mRNA*_ were then computed.

Finally, the dysregulated score *S* of an LMM-CT was calculated by combining the node score, edge score, and the influence score as follows

8 $$S = \mathop{\sum}\nolimits{_{{{\rm{node}} \in {\rm{LMM}}\_{\rm{CT}}}}\,{\rm{DotScore}}} + \mathop{\sum}\nolimits{_{{{\rm{edge}} \in {\rm{LMM}}\_{\rm{CT}}}}\,{\rm{EdgeScore}} + {\rm{InfluenceScore}}}$$

In addition, the statistical significance of each LMM-CT was estimated using a permutation test. RNA labels of samples were randomly shuffled to construct a random LMM-CT, and the LMM-CT score was then recomputed. The above procedure was repeated 10,000 times, and the null distribution for the LMM-CT score was subsequently obtained. For each LMM-CT, an empirical *P*-value was defined as the proportion of randomly obtained LMM-CT scores larger than the real LMM-CT score, as shown in Eq. (9)

9 $$P - {\rm{value}} = \left( {{{{{Number}}}\;{{of}}}\;S_{{{{\mathrm{random}}}}} \,>\, S} \right)/10,000$$

In this study, LMM-CTs with a *P*-value < 0.05 were selected as the dysregulated LMM-CTs.

### Construction of time-ordered background ceRNA networks in NF

By combining the LMM-CTs in NF identified above, a ceRNA network was obtained. The largest net component was defined as the background ceRNA network in NF, which included 10,835 LMM-CTs consisting of 181 lncRNAs, 143 miRNAs, and 2810 mRNAs. To investigate disease progression, a previous method was employed to construct TO-BCeN in NF [38]. Initial nodes were selected based on the following hypothesis: To maintain the organism in the normal state, the initial nodes in the TO-BCeN needed to be relatively stable, otherwise, their dysregulation would lead to the instability of the entire TO-BCeN. Therefore, nodes having the lowest dysregulated scores were chosen as the initial nodes. Since a node may be involved in multiple LMM-CTs, the dysregulated score of a node was defined as the mean of the dysregulated scores of the LMM-CTs in which it participated. Using the initial nodes, a BFS on the NF background ceRNA networks was employed, and the TO-BCeN was constructed. To facilitate subsequent analysis, a single gene-based network was converted into an LMM-CT-based network; that is, if one node in an LMM-CT first appeared in a time-ordered level, the LMM-CT was considered as being in that level. Simultaneously, the LMM-CT was deleted in the following levels.

### An LMM-CT-based dysregulated score of each patient

In the present study, a method has been presented for identifying disease-associated dysregulated LMM-CTs based on the ceRNA mechanism. To further investigate the classification efficiency of these dysregulated LMM-CTs in distinguishing patients with ICM from those with DCM, a multiple regression model was proposed to yield a dysregulated score for each patient according to the ceRNA hypothesis. For one LMM-CT, the mRNA expression level was presumed to be affected both by the expression level of the miRNA and the interaction between the miRNA and lncRNA; therefore, the multiple regression was modeled according to Eq. (10)

10 $${{{{Exp}}_{{{mRNA}}}}} = a_1 \times {{{{Exp}}_{{{miRNA}}}}} + a_2 \times {{{{Exp}}_{{{miRNA}}}}} \times {{{{Exp}}_{{{lncRNA}}}}} + c$$

where Exp denotes the gene expression level, *a*_1_ and *a*_2_ represent regression coefficients, and *c* denotes the error. It is worth noting that the effects of interactions among multiple LMM-CTs were not taken into consideration for the sake of maintaining the simplicity of the analysis.

As mentioned above, it was assumed that LMM-CTs exist in NF individuals and that a normal biological stable state is maintained. Therefore, it would be possible to obtain *a*_1_, *a*_2_, and *c* under normal conditions using NF samples. For a patient and a given LMM-CT, an estimated mRNA expression value could be calculated by Eq. (10) using corresponding miRNA and lncRNA expression information. The LMM-CT-based dysregulated score (DS) of a patient was defined as the difference between the estimated mRNA expression and the real expression levels, according to Eq. (11)

11 $${{{{DS}} = {{Exp}}_{{{mRNA}}}^{{{case}} \ast } - {{Exp}}_{{{mRNA}}}^{{{case}}}}}$$

where ${{{{Exp}}_{{{mRNA}}}^{{{case}} \ast }}}$ and ${{{{Exp}}_{{{mRNA}}}^{{{case}}}}}$ denote the estimated and real mRNA expression levels, respectively.

### Identification of diagnostic biomarkers

In this study, two types of diagnostic biomarkers were identified: one was for distinguishing patients with ICM/DCM from the controls, and the other was for differentiating ICM from DCM patients.

A random forest supervised classification model was used to select genes that were strongly associated with the disease diagnosis [39]. At each step, an important score was calculated for each gene by using a permutation test, and the genes that fell into the category of having the lowest one-third important scores were discarded. In this manner, those genes that were most strongly associated with the diagnosis were reserved. Finally, classification accuracy for all combinations of these remaining genes was computed using the support vector machines (SVM) classification algorithm, and the optimal diagnostic biomarkers were identified, taking into account the balance between classification accuracy and the number of genes.

Random forest and SVM algorithms were performed using the R packages “randomForest” and “e1071”, respectively. Classification accuracy and the AUC based on the LOOCV were applied to measure the performance.

## Supplementary information

Supplementary legends

Supplementary Table S1

Supplementary Table S2

Supplementary Table S3

Supplementary Table S4

Supplementary Table S5

Supplementary Table S6

## Data Availability

The datasets generate or analyzed during this study are included in this published article and its Supplementary information files.

## Code availability

The source code of the algorithm for identification of dysregulated LMM-CTs in disease is available from the corresponding author upon request.
