# Supplementary material for: Time-ordered dysregulated ceRNA networks reveal disease progression and diagnostic biomarkers in ischemic and dilated cardiomyopathy
Source: Cell Death Discov. 2021 Oct 16;7:296. doi: 10.1038/s41420-021-00687-7 (PMC8520530; doi:10.1038/s41420-021-00687-7)
Supplement: Supplementary file 1 — Supplementary legends [file 41420_2021_687_MOESM1_ESM.docx]

# Supplementary legends

**Supplementary Table S1. The identified dysregulated LMM-CTs in ICM and DCM.**

**Supplementary Table S2. Summary of LMM-CTs at different time-ordered levels in the TO-BCeN.**

**Supplementary Table S3. Significantly enriched GO BP terms of mRNAs in ICM dysregulated LMM-CTs at different time-ordered levels.**

**Supplementary Table S4. Significantly enriched GO BP terms of mRNAs in DCM dysregulated LMM-CTs at different time-ordered levels.**

**Supplementary Table S5. Summary of clinical information of the samples used in the present study.**

**Supplementary Table S6. Summary of the datasets used in the present study.**
