# Supplementary material for: Time-ordered dysregulated ceRNA networks reveal disease progression and diagnostic biomarkers in ischemic and dilated cardiomyopathy
Source: Cell Death Discov. 2021 Oct 16;7:296. doi: 10.1038/s41420-021-00687-7 (PMC8520530; doi:10.1038/s41420-021-00687-7)
Supplement: Supplementary file 7 — Supplementary Table S6 [file 41420_2021_687_MOESM7_ESM.docx]

## Supplementary Table S1. Summary of the datasets used in the present study.

| **GEO ID** | **Data type** | **Data set** | **No. of ICM** | **No. of DCM** | **No. of NF** |
| --- | --- | --- | --- | --- | --- |
| GSE46224 | Sample matched  RNA-seq and miRNA-seq | Training set | 8 | 8 | 8 |
| GSE53080 | miRNA-seq | Test set | 14 | 22 | 10 |
| GSE116250 | RNA-seq | Test set | 13 | 37 | 14 |
| GSE1145 | microarray | Test set | 31 | 27 | 11 |
